# Supplementary figures and images for: Body composition among Malawian young adolescents: Cross-validating predictive equations for bioelectric impedance analysis using deuterium dilution method
Source: PLoS One. 2023 Apr 12;18(4):e0284158. doi: 10.1371/journal.pone.0284158 (PMC10096513; doi:10.1371/journal.pone.0284158)

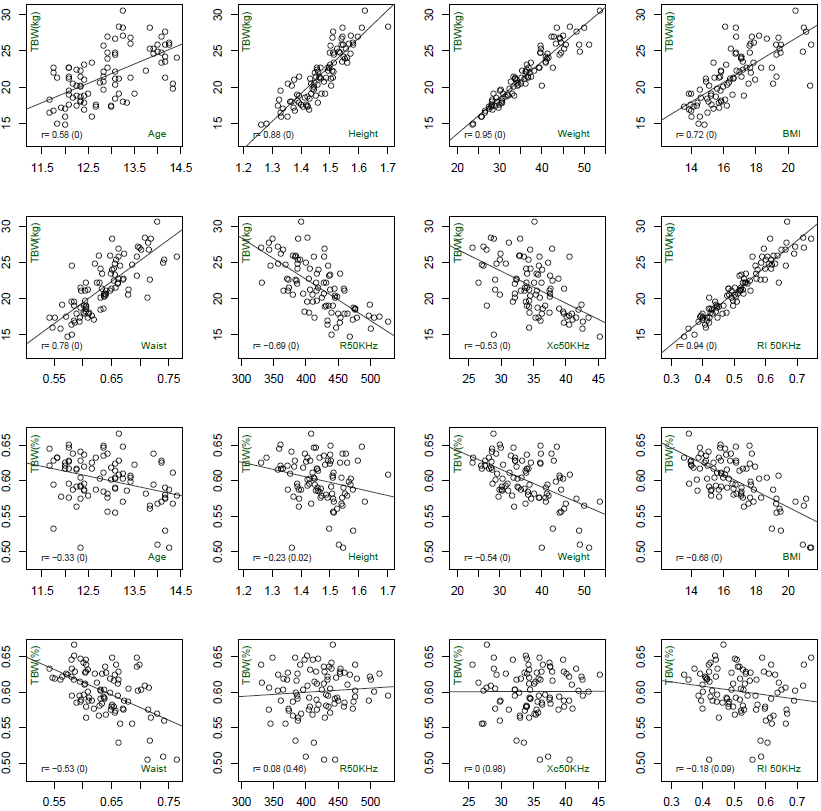

Supplement: S1 Fig — Estimated regression lines plotted. Correlation coefficient r (with p-value) listed on bottom left corner. R = resistance, Xc = reactance, RI = resistance index, TBW (kg) = total body water in kilograms, TBW(%) = total body water as percentage of weight. (TIF) [file pone.0284158.s001.tif]

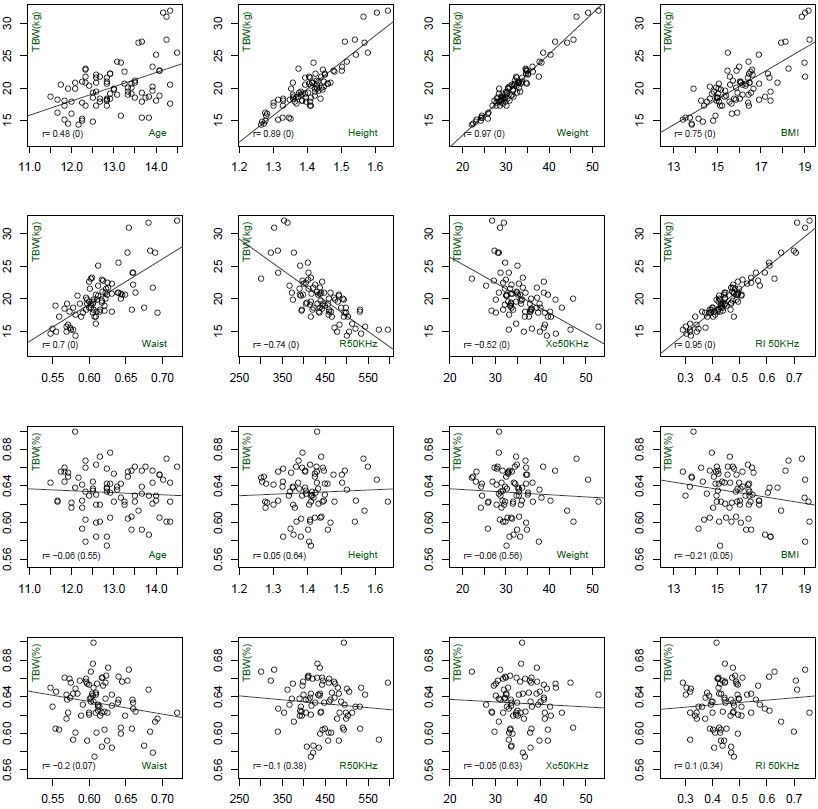

Supplement: S2 Fig — Estimated regression lines plotted. Correlation coefficient r (with p-value) listed on bottom left corner. R = resistance, Xc = reactance, RI = resistance index, TBW (kg) = total body water in kilograms, TBW(%) = total body water as percentage of weight. (TIF) [file pone.0284158.s002.tif]
