# Supplementary material for: Body composition among Malawian young adolescents: Cross-validating predictive equations for bioelectric impedance analysis using deuterium dilution method
Source: PLoS One. 2023 Apr 12;18(4):e0284158. doi: 10.1371/journal.pone.0284158 (PMC10096513; doi:10.1371/journal.pone.0284158)
Supplement: Attachment — Submitted filename: renamed_194d4.docx [file pone.0284158.s005.docx]

Manuscript PONE-D-2021-31743-R1 Näsänen-Gilmore et al.

**Comments by the editorial office.**

1. Please ensure that your manuscript meets PLOS ONE's style requirements, including those for file naming. The PLOS ONE style templates can be found at
   <https://journals.plos.org/plosone/s/file?id=wjVg/PLOSOne_formatting_sample_main_body.pdf> and 
   <https://journals.plos.org/plosone/s/file?id=ba62/PLOSOne_formatting_sample_title_authors_affiliations.pdf>.

*RESPONSE: Done.*

2. Please provide additional details regarding participant consent. In the ethics statement in the Methods and online submission information, please ensure that you have specified what type you obtained (for instance, written or verbal, and if verbal, how it was documented and witnessed). If your study included minors, state whether you obtained consent from parents or guardians. If the need for consent was waived by the ethics committee, please include this information.

*RESPONSE: We have added more detail, and the text reads now: “We obtained an assent from each participant and a written informed consent from their guardian on the day of the testing. The consent form was read and explained to the guardian in their preferred language (chewa or yao) and any questions were answered before* *the guardian signed the consent form by signature or thumbprint.”*

3. Please include a complete copy of PLOS’ questionnaire on inclusivity in global research in your revised manuscript. Our policy for research in this area aims to improve transparency in the reporting of research performed outside of researchers’ own country or community. The policy applies to researchers who have travelled to a different country to conduct research, research with Indigenous populations or their lands, and research on cultural artefacts. The questionnaire can also be requested at the journal’s discretion for any other submissions, even if these conditions are not met.  Please find more information on the policy and a link to download a blank copy of the questionnaire here: <https://journals.plos.org/plosone/s/best-practices-in-research-reporting>. Please upload a completed version of your questionnaire as Supporting Information when you resubmit your manuscript.

*RESPONSE: Please see attached form.*

4. Thank you for stating the following financial disclosure:
    [The original LAIS study was supported by grants from the Academy of Finland (grants 79787 and 207010 to PA; aka.fi), the Foundation of Pediatric Research in Finland (lastentautientutkimussaatio.fi ; PA), and the Medical Research Fund of Tampere University Hospital (PA). This study was supported by the Academy of Finland (EK; aka.fi), Finnish Medical Foundation (Finska Läkaresällskapet) (EK), Foundation for Pediatric Research in Finland (EK, lastentautientutkimussaatio.fi), Novo Nordisk Foundation (EK, novonordiskfonden.dk), Signe and Ane Gyllenberg Foundation (PNG, EK; gyllenbergs.fi) and Sigrid Juselius Foundation (EK; sigridjuselius.fi).]
    
   Please state what role the funders took in the study.  If the funders had no role, please state: "The funders had no role in study design, data collection and analysis, decision to publish, or preparation of the manuscript." 
   If this statement is not correct you must amend it as needed. 
   Please include this amended Role of Funder statement in your cover letter; we will change the online submission form on your behalf.

*RESPONSE: Thank you. The statement is correct and is included in the cover letter.*

5. In your Data Availability statement, you have not specified where the minimal data set underlying the results described in your manuscript can be found. PLOS defines a study's minimal data set as the underlying data used to reach the conclusions drawn in the manuscript and any additional data required to replicate the reported study findings in their entirety. All PLOS journals require that the minimal data set be made fully available. For more information about our data policy, please see <http://journals.plos.org/plosone/s/data-availability>.

Upon re-submitting your revised manuscript, please upload your study’s minimal underlying data set as either Supporting Information files or to a stable, public repository and include the relevant URLs, DOIs, or accession numbers within your revised cover letter. For a list of acceptable repositories, please see <http://journals.plos.org/plosone/s/data-availability#loc-recommended-repositories>. Any potentially identifying patient information must be fully anonymized.

Important: If there are ethical or legal restrictions to sharing your data publicly, please explain these restrictions in detail. Please see our guidelines for more information on what we consider unacceptable restrictions to publicly sharing data: <http://journals.plos.org/plosone/s/data-availability#loc-unacceptable-data-access-restrictions>. Note that it is not acceptable for the authors to be the sole named individuals responsible for ensuring data access.

We will update your Data Availability statement to reflect the information you provide in your cover letter.

*RESPONSE: Our data are sensitive health data and we do not have consent from the study participants to make the date publicly available. This is also indicated in the cover letter.*

6. Your ethics statement should only appear in the Methods section of your manuscript. If your ethics statement is written in any section besides the Methods, please move it to the Methods section and delete it from any other section. Please ensure that your ethics statement is included in your manuscript, as the ethics statement entered into the online submission form will not be published alongside your manuscript.

*RESPONSE: The ethics statement is currently in the Methods section.*

7. Please upload a copy of Figures 2 and 3, to which you refer in your text on page 12. If the figure is no longer to be included as part of the submission please remove all reference to it within the text.

*RESPONSE: We apologise this regrettable omission. The Figures are now included.*

*Please note that the row numbers in the responses refer to the track changes version.*

**Reviewer #1:**

A generally clearly written manuscript that describes an ostensibly well-conducted study. Some points for the authors to consider.

Line 66 onward. This is a simplistic description of BIA but not inaccurate. The authors might consider describing the differences between single, multi-frequency and BIS. I note that they used what is essentially a BIS device (19 frequencies) but BIS analysis was not used, preferring empirical equation generation. Why not? This could be readily performed since the authors state that all raw data were obtained (line 127). The authors could then test whether their equations perform better or worse than BIS prediction (see publications by Lyons-Reid for further information - in this regard some of the citing references are rather old and more up-to-date reference could be used).

*RESPONSE: We agree that the data collected with Seca m515 device would have made BIS analysis possible. However, the most important added value of BIS analysis would have been the distinction between the intracellular and extracellular water components. The aim of our study was to create and cross-validate prediction equations for total body water using deuterium dilution as the reference method. Deuterium dilution gives an estimate of total body water (based on two-compartment model of body composition) and does not distinguish between intracellular and extracellular water. For this reason this distinction is irrelevant for the present study.*

*The papers by Lyons-Reid et al. focus on infants and small children. We do not currently see a point to be referred to in these papers but are happy to reconsider this if the reviewer has a specific request.*

Line 68 "Resistance and reactance depend on water content of the tissue and, with appropriate prediction equations," is not quite correct. Reactance Xc is determined by the capacitive nature of cell membranes. BIS does not use empirical predictive equations

*RESPONSE: We have revised the sentence which now reads “Resistance and reactance depend, among other determinants, on water content of the tissue and, with appropriate prediction equations, can therefore be used to calculate total body water (TBW).”*

Line 73 onward. I agree that DD is a reference method for TBW but do not agree that it is difficult in children and adolescents. There are many studies attesting to its use in this population and, indeed, the IAEA, whose protocols were followed promote and support its use in children. Furthermore, if absolutely necessary fluid intake is allowed and can be corrected for (see Schoeller's chapter on "Hydrometry" in "Human Body Composition" by Heymsfield.

*RESPONSE: We agree that our wordings have not been optimal. We have revised the sentence as follows: “However, this method is labour-intensive and lengthy to perform [12] and does not allow free intake of liquid during the procedure, and hence requires some effort to carry out on children or young adolescents.”*

Line 79. There may not be other studies validating using DD but there are papers that use DXA which is again an accepted reference method , e.g. van Zyl et al. I suggest that you make this point.

*RESPONSE: Thank you for bringing up this. Indeed the Zyl et al studies use DXA as reference. We have added a reference to this study in the manuscript.*

Lines 82, 92 and elsewhere please check for inappropriate capitalization.

*RESPONSE: Thank you. This has been corrected.*

Line 124. BIA is a technique that requires careful standardisation of the protocol. Please provide full details here. See papers by Brantlov for guidance on reporting standards.

*RESPONSE: On lines 149 to 152, we have added a more detailed description of the measurement procedure.*

The DD method is appropriately described but details of quality control procedures are required as discussed in the IAEA monographs on the technique.

*RESPONSE: We have added the requested detail on lines 162-195.*

Data analysis The initial regression strategy seems to be rather shotgun - 23 variables! For example, why resistance at 5 frequencies but Xc at only 50 kHz (note kHz NOT KHz)? Why these particular frequencies? What is the rationale for including R and RI in the prediction equation 1 in Table 4? BIA theory clearly shows that conductive volume (e.g. TBW) = rho H^2/R NOT simply R. R alone is going to be raled to length and cross-section of the conductive volume which relates to TBW but will not be independent of RI. Simply using VIF to eliminate (choose) variables is inappropriate, select on strong theoretical underpinnings.

*RESPONSE:*  *Our approach was to first use a data-driven algorithm to select the best performing predictive equation without reference to theory or previous studies. We then checked that the resulting equation did not exclude variables that would a priori be considered essential and was not in contradiction to theory or previous studies. In our judgement these requirements was satisfied by each equation that we present. As expected, RI (=H^2/R) was always the strongest single predictor. The best equation to predict TBW(kg) in equation 1 in Table 4 also included R as this improve predictive performance and we do not see any valid reason to exclude R just because RI is also in the equation. We fully agree that R alone may not be an optimal predictor; in the manuscript it is not included without RI.*

*We agree that selecting predictors for the BIA-equation on theoretical grounds and previous studies probably leads to best predictive models. However, considering that in our setting age group and geographical setting are new and previous studies are rare, we started from a large set of potential variables and then verified that we end up in a reasonable model also on theoretical grounds. We have now added text emphasizing this point in the Discussion (lines 331-339).*

*The data driven algorithm initially included all 23 variables as potential predictors. Large number of candidate predictor variables did not harm the variable selection process. On the contrary, in predictive modelling, considering all potential predictors is important and, when a proper algorithm is applied in variable selection (for example utilizing cross-validation techniques), avoidance of overfitting and exclusion of mutually correlated predictors is a built-in feature of the variable selection algorithm. Therefore, we did not need to use VIF-values and we do not see the initial number of candidate predictors as problematic.*

*Nevertheless, we have reduced the number of potential reactance and resistance variables before actual variable selection, please see our response to the next question.*

Line 189. I do not understand this. Magnitude of R is clearly frequency dependent, the basic BIA Cole model shows this and it is readily observed in practice - look at your R data for the 19 frequencies. I do not see the theoretical rationale for using Xc at different frequencies. This is getting close to a BIS-style frequency-dependent analysis but does not appear to be justified here. This is important since it gets to the heart of your equation generation and needs better justification.

*RESPONSE: Our wording on Line 189 (now lines 220-221) was in fact a regrettable error. We thank the referee for pointing this out. The values are not at all similar. We have now corrected this.*

*We meant to say that the R-values are not similar, but all pairs are linearly almost precisely dependent (i.e. all pairwise correlations are almost exactly 1). Therefore, in regression modelling, the set of R variables are essentially one single predictor.*

*Accordingly, when all R (resistance) and Xc (reactance)-values (and corresponding RI values H^2/R) are included in the candidate variable list, at most one of the frequencies corresponding to the correlated group of variables is selected for the same predictive model and, apart from this, the equations are essentially the same we obtain with the reduced variable set. Therefore, to simplify presentation and to avoid the essentially same variable at varying kHz-versions appearing in different equations, we decided to include only one of the R-frequencies in the list of candidate variables. 50Hz was selected as it is common in other studies.*

*The reactance variables with different frequencies are also linearly dependent but not quite as strongly as resistance variables. Therefore, 5 levels (1,15,50,200,1000 kHz) was deemed sufficient as candidate predictors. However, eventually none of the reactance variables was selected as a predictor by our modelling procedure.*

**Line 194 onward. The K-fold validation process is OK to get an idea of overall model error but inevitably the mean values are equivalent to the overall population mean, hence you essentially 0 biases seen in the LOA analysis Line 272 on. The "true bias" should be assessed by using an independent cross-validation group. This can be undertaken with your data by splitting the group into two sex-stratified cohorts of say 2/3 and 1/3. Generate the equation in the 2/3 group and test in the 1/3 using LOA and correlation (use concordance not Pearson). This should be done, at present your bias values are meaningless.**

*RESPONSE: The referee is correct that in our study the bias is bound to be close to 0. One can calculate bias using a single randomly selected cross validation group based on a 2/3 and 1/3 split, but this value would be quite arbitrary, depending on the random split. Our bias is the average bias from several repeated splits (see below), and it can be expected that on average there is no systematic bias.*

*We argue that bias would be more of interest only if the equation is applied to a genuinely new data set from a separate study and not to a randomly selected hold-out set. In our opinion, the only useful measure of the performance of the predictive equation in our setting is the out of sample RMSE-value, i.e. the estimated (external) expected error of the predicted value with respect to the unknown true value for a single new individual (i.e. individual that was not used in modeling).*

*Unless data is very large, a single split of the cohort into 2/3 training data and 1/3 testing data is not accurate for a reliable prediction error estimate of variable selection. Using another split will easily produce different results both in model selection and in out of sample error estimation. We have now added a sentence and references (Molinaro et al, 2005, Harrell, 2015) which highlight these problems with the single split approach. Therefore, in our paper the random split was done several times (in fact 1000 times) using the 10-fold cross validation approach and we do not rely on a single random split.*

*We stress that while our approach involved the 10-fold cross-validation also as a variable selection approach, an entirely separate cross-validation was used for external error estimation (the “outer layer” in nested cross validation). The data which was used in model building was never used for error estimation. An independent hold-out data was always used for this purpose.*

*In short we used 10% as a hold-out data, then built the model using 90% of the data and within the 90% used a separate (“inner layer”) cross-validation to find the best model (with the hold-out data omitted) and then predicted the hold-out data. This was done 1000 times using a 10-fold cross validation framework that was repeated 100 times (nested cross validation approach).*

*Accurate estimation of the out of sample RMSE and letting all observations contribute to model selection, via repeated splitting of the data to modelling and hold-out sets, is an important aspect of our paper. We have now tried to modify the wording of the manuscript in Methods and Discussion to clarify this.*

**Line 212 and equation b. Given the relationship between RI and TBW above, equations should not be generated in relative (%) terms. Delete this.**

*RESPONSE: Equations in relative terms are not unusual in BIA studies (e.g. Houtkooper(1989)* [*Validity of bioelectric impedance for body composition assessment in children*](https://journals.physiology.org/doi/abs/10.1152/jappl.1989.66.2.814)*; an many other references). We think they are particularly illuminating in our study.*

*In our case there is considerable variation in the weight of the adolescents. Therefore, predicting TBW(kg) is seemingly very easy and part of the high predictive power of our TBW(kg) equation is actually only due to the large variation in person’s weight. Therefore, showing how well (or poorly) TBW(%) can be predicted is important.*

*We think that the wide range in weight is the reason why, in our data, using the TBW(%) equation and transforming these %-wise predictions to kg produces better predictions for TBW(kg). We have added a sentence regarding this in Discussion.*

Results are generally well presented with the caveats noted above. But the LOA are not reflective of "true" LOA; the same sample has been used to generate the equation and to test the equation - this is circular. The split sample test described above should be used.

*RESPONSE: We have now modified the manuscript to make it clear that the prediction error is estimated from independent data not used in model building. Please see the response above.*

The "true" LOA are likely to be somewhat larger. For the LOA as given this was around +/- 1.4 kg. Since TBW was around 20 kg this means that the LOA are around +/- 7% Is this clinically acceptable? See Ward in EJCN for a discussion on this point. This point needs to be discussed as well as the overall error (lines 304 on) since it represents the predictive power for an individual rather than for a population (the bias and associated error).

*RESPONSE: We agree that the viewpoint of individual-level prediction merits being discussed in the paper. However, we also point out that the main rationale of the study is to allow a more reliable measurement of body composition in scientific studies on a group level. As suggested, we have added a statement regarding the individual-level prediction errors on Lines 376-380.*

*Regarding predictive power for an individual vs bias, see response above.*

Discussion

Line 288 on the authors appear to rely on the statistical approach to parameter selection. This is important but is not the most important. Parameters should only be included on justifiable biological model grounds not simply BIC or AIC criteria. I repeat that the authors need to justify physiologically and congruent with the BIA model the choice of predictive parameters.

*RESPONSE: We agree that this is an important point. Referring to the response above, while the variable selection approach was initially not based on theoretical grounds, the resulting equations are well justifiable on biological grounds. Resistance index, sex, weight, height, BMI and resistance are commonly found important in equations presented in bioimpedance studies.*

**Please provide the LOA plots as supplementary data (after the re-analysis as requested).
Line 299 This needs to address the point made above that K-folding does not highlight true predictive error for individuals and does not provide a true bias estimate.**

*RESPONSE: We apologize that the LOA plots (Figs 2 and 3) were accidentally not included in the original submission apparently because of confusion at the file upload stage. The error rate we report is obtained independently of model fitting (using an “outer” K-folding that is separate from variable selection K-folding), as described above, and error estimates are for independent (true) predictive errors.*

**The seca device used here provides its own predictions of TBW. How do these compare to the the DD and the predictive equations output? It is important that this be tested along with some similar published equations for cognate groups e.g. the refs cited by the authors. This is important since as the authors allude equations are population specific. Also they should test against the fundamental BIS approach which is purported to not be pop-specific.**

*RESPONSE: We are unable to meet this request because the Seca m515 device does not provide TBW predictions for children or adolescents. Moreover, we did not find data originating from a low resource setting setting corresponding to the same age group as ours that would have been available to test our equations in a comparable setting. As to the BIS approach, please see our response to the first comment above.*

**In conclusion, the study data collection appears sound but the data analysis should be expanded and appropriate analyses conducted.**

*RESPONSE: We thank the referee for careful reading of the manuscript and hope that the concerns would be met by the clarifications we have done in the manuscript text and the responses above. Our understanding is that the expanded analyses that the referee requires are essentially included in the manuscript as it is, albeit using a somewhat different approach than suggested by the referee (we utilise several hold-out sets instead of just one). We hope that we have understood this correctly and will be happy to reconsider this if so requested by the referee or editor.*

*We acknowledge that our data-driven predictive model building approach is somewhat different and computationally more elaborate than typically applied in BIA studies. However, we claim that our approach results in a carefully selected predictive model and a more reliable prediction error rate estimate than using a less computationally intensive approach. Our aim was to apply predictive modelling techniques somewhat in the same fashion as they are nowadays very widely and effectively applied in engineering and commercial applications (c.f. several of the references related to predictive modelling).*

**Reviewer #2: Comments to the authors**

In this study, the authors derived a predictive equation to estimate fat-free mass from total body water measured by BIA in children (11-14 years) in Malawi using isotopic dilution as the gold standard method. We are lacking inexpensive, easy to implement and reliable methods to assess body composition in African populations, especially in children. This study is therefore important as it is filling a gap in knowledge and will allow to conduct further nutrition studies in these vulnerable populations that require specific public health guidelines and actions.

Although we appreciate the goal of the study, the current scientific approach presents few weaknesses that need to be addressed. Below we are suggesting some edits and alternative approaches to the authors hoping they will allow to improve the quality of the manuscript.

**Major comments
It is unclear why the predictive equations, RMSE, p-values etc are different for TBW expressed in % and in kg, when one is the direct relationship of the other (Table 3). The methods/models used to derive TBW in kg and TBW in kg calculated from TBW in % is also confusing. Please further explain your approach and justify why those equations are different.**

*RESPONSE: We have now clarified the description of the derivation of equations to be more transparent. In brief, the TBW(kg) predictions using TBW% are obtained simply by multiplying the TBW%-predictions for each person, as obtained from the equation derived for TBW%, by that person’s weight.*

*In our case there is considerable variation in the weight of the adolescents. Therefore, predicting TBW(kg) is seemingly very easy and part of the high predictive power of our TBW(kg) equation is actually only due to the large variation in person’s weight. Therefore, showing how well (or poorly) TBW(%) can be predicted is important.*

*We think that the wide range in weight is one key reason why, in our data, using the TBW(%) equation and transforming these %-wise predictions to kg produces better predictions for TBW(kg). We have added a piece of text regarding this in Discussion (lines 350-355)*

**As mentioned by the authors, the fact that all the children were not fasted and had an empty bladder is major limitation as it is not following the standard protocol for these measurements. Please consider running the analysis in the children who were both fasted and with an empty bladder and then compare with your current equations. If the two are close, please provide the equations in the subgroup as supplemental material. If they are different, please only provide the equation derived with the kids who complied with the instructions.**

*RESPONSE: We agree that non-standardised fasting is a limitation. However, this limitation also reflects practical circumstances in conducting studies in low-resource settings. Moreover, due to small sample sizes, results for analyses in subgroups the referee suggests would involve a lot of uncertainty. Particularly for the “fasted and empty bladder in the morning”-group, the difference is not clear as there are only 52 such children.*

*When we considered separate models for the fasted group (N=90) and for those who did not fast (N=88), we noticed that the prediction error is higher and the form of the best performing equation is more uncertain for the non-fasted group.*

*However, as seen in Table 4, fasting (yes/no) was not selected as a predictor for TBW indicating that separate models for the subgroups involve a lot of uncertainty. For the fasted group, the joint model in Table 3 performs almost as well as the best predictive model derived separately for that group and the best predictor variables are the same. For the non-fasted group, the joint model is much better. Therefore, we think that equations in Table 4 should be used for both groups. In addition, due to the uncertainties in separate equations and the fact that reported fasting status itself is also uncertain, to avoid confusion, we would much prefer not presenting separate results here. We discuss fasting as a limitation in the limitations paragraph (lines 390-391).*

**We suggest to only use height and weight in the equations and not BMI as it is a ratio of the two first variables.**

*RESPONSE: We would like to point out that height and weight carry much of the same information, because weight is a summary indicator of all three spatial dimensions of body size and height indicates one of these body size dimensions. The rationale of using BMI as a measure of body size is to have a measure that indicates the two other dimensions and accordingly is less dependent on height than weight is. Consistent with this, among our participants the correlation coefficient between height and weight is 0.87, between weight and BMI 0.84, and between height and BMI 0.47. As expected based on this, our best predictive equation for TBW(%) includes height and BMI. Using just weight instead of BMI would result in inferior predictions. Height being also a component BMI indicates that the dependency on height is not linear and that this nonlinearity is important to be taken into account. In our view, transformations (and interactions) of the original variables can in general be valid potential predictors alongside with the original variables. BMI is a prime example of such a transformed variable.* **Please consider discussing the variance, mean bias and mean prediction error of your model in light of the clinically significant differences/changes for fat-free mass in children to know whether or not this method could be powerful enough in a clinical setting.**

*RESPONSE: We suggest that bias would be more of interest only if the equation is applied to a genuinely new data set from a separate study (and not to a randomly selected hold-out sets as we do). In our opinion, the only useful measure of the performance of the predictive equation in our setting is the out of sample RMSE-value, i.e. the estimated (external) expected error of the predicted value with respect to the unknown true value for a single new individual (i.e. individual that was not used in modeling).*

*We agree that it is important to make clear whether the predictive equations would be useful in a clinical setting, e.g. in an individual level. Indeed the main rationale of our study is to enable a more reliable measurement of body composition in scientific studies on a group level. As suggested, we have added a statement regarding the individual-level prediction errors on Lines 376-380.*

As the hydration factor is about 0.75, an error of magnitude 0.7kg in TBW(kg) translates to roughly 1kg average error in FM.

RESPONSE: This is correct. We have now extended the discussion on the mean prediction error and 95% limits of agreement (lines 362-370) by illustrating these for TBW(kg) in absolute (kg) and standardized (SD) units. We believe this illustrates simply but adequately the degree of error in the analysis.

Although we understand that the BIA from Semca is likely more cost-effective than the use of deuterated water because of the cost of the samples analysis, it is not necessarily an inexpensive method. The purchase of this device cannot be afforded by all medical settings and its access may likely be restricted major clinics. It would have been interesting to also compare those two methods to another cheap method such as the skinfold thickness method. Even if much more inaccurate, such a method could be interesting for large epidemiological studies, especially in ultra-rural and remoted areas. Other devices such as portable Tanita BIS could also be easier to use in such environments. Although we understand the authors derived an equation using a device they had access to, it does not necessarily address the need for a method to estimate body composition that is easy to implement, cheap and accurate. We would like to invite the authors to discuss those considerations.

*RESPONSE: Indeed our aim was to create and cross-validate predictive equations for body composition / total body water measurement by bioimpedance, using deuterium dilution as a reference method. Validation or comparison with other methods such as skinfold thickness would in our opinion be a subject of a separate study. We agree that the cost of the device may be prohibitive for some studies and that more portable devices would be suitable in studies conducted in varying geographical locations. We have added a sentence on this in the limitations paragraph (lines 392-394).*

Please clearly specify the inclusion/exclusion criteria of the study participants.

*RESPONSE: As described in the Materials and Methods section, the participants were chosen randomly among participants who had attended an ongoing follow-up of the LAIS cohort. There were no exclusion criteria. We have added some detail in the description of this procedure (lines 197-119).*

Please proof-read English.

*RESPONSE: We have proof-read the English in the manuscript carefully.*

Introduction
Lines 61-62 : BMI being used as an index of overweight and obesity, it is logical that BMI increases along with the obesity epidemic progresses. Please rephrase this sentence.

*RESPONSE: We have removed the words “Along with obesity epidemic, ….” (line 61).*

Line 62: Obesity is also due to malnutrition. Did you mean “undernutrition?

*RESPONSE: Thank you for pointing this out. We have replaced “malnutrition” by undernutrition”.*

Line 65: Consider adding a sentence on the current needs, i.e. developing an accurate, simple, cheap/affordable and easy to administer method that can be used to assess body composition in large population studies to better delineate future public health guidelines.

*Response: We have added a brief sentence on lines 70-71.*

Line 71: Remove “a” prior to “healthy adults”

*RESPONSE: Done.*

Line 72: Replace total body weight by total body mass.

*RESPONSE: We would prefer the use of “weight” and “total body weight” in this context. This is widely used in the literature, including validation studies in Africa (references 14-17).*

Line 73-75: This method is also pricy, requires specific devices (IRMS or infrared spectroscopy) for samples analysis that are possessed by only few labs around the world, that are very expensive and need specific expertise.

*RESPONSE*: *While we think that the analysis is done in more than a few labs around the world, we agree that there is a substantial cost involved. We have added the words “relatively costly” on line 76.*

Line 78-79: The gap in knowledge is here implicit but needs to be clearly described like it is nicely done in the abstract.

*RESPONSE: We believe that the gap in knowledge should be apparent from the text on lines 85-87.*

Line 81: It may not be needed to provide the details of the BIA device in the introduction section.

*RESPONSE: We would prefer to state the brand of the device as the prediction equations are device-specific.*

The scientific premise of the study would benefit for being a bit more substantial; for example, it is also important to justify the study population, children and young adolescents. Why an age range comprised between 11 and 14 years old? We also invite the authors to explain why they believe BIA is more adequate than other available methods for measuring their body composition (e.g., skinfold thickness, BIS, others).

*RESPONSE: We believe we have justified the importance of studying youth on lines 69-70. As the aim of this paper is to describe creation and cross-validation of prediction equations for bioimpedance analysis, and not comparison of methods to measure body composition, we would like to refrain from more extensive discussion on this topic. Comparisons are available in the literature, including the references of this paper.*

Line 86: Were the informed consent signed or orally obtained? In a population where a large portion of the population may be illiterate, it is important to provide more information about the methods for consent obtention.

*RESPONSE: Thank you. On lines 101-102, we have added a sentence “The consent form was read to the guardian in their preferred language (yao or chewa) and any questions were answered before signing the consent form.”*

M&M

Line 95: Sample size of mothers? Sample size of the kids who were potentially available for this study?

*RESPONSE: We selected randomly 120 boys and 120 girls from a sample of 728 children. We have added some more detail to the description on lines 117-119. We do not think that the sample size of mothers is relevant for the present study.*

**Line 118: How many kids reported not being fasted on the study day?**

*RESPONSE: 38 boys and 52 girls. Total 90. Table 2 in the manuscript had a regrettable error in these frequencies, which were erroneously for those who did not fast. This has now been corrected.*

Lines 120-121: It is not clear why it was not asked to all kids to empty their bladder prior to dosing.

*RESPONSE: This was indeed an unfortunate omission and has been mentioned as a limitation on line 391.*

Body composition by bioimpedance: please provide more information about the measurement including data analysis to obtain body composition, placement of the electrodes, etc.
It would have been interesting to take the measurement in duplicate.
It is unclear why weight was measured at the beginning and at the end of the experiment, and why was it taken into account? To what the weight change over few hours can be attributed? Can it be attributed to the intra-individual precision of the BIA? This information on the device should be provided.

*RESPONSE: On lines 149-152, we have added more detail on the measurement procedure, including the placement of the electrodes. We are not sure what kind of detail the reviewer requests as to data analysis, which is described in detail on lines 188-234. Weight change during the process was included to provide an estimate of possible isotopic fragmentation through liquid evaporation, but was not included in the predictive equations by the modelling procedure.*

Body composition by deuterium dilution
Line 151: Add “post-dose” after “the 3 hour and 4 hour”

*RESPONSE: Done.*

Please add a reference showing that the method using saliva samples has been validated against the one with urinary samples.

*RESPONSE: on lines 156-157, we have added a sentence “Deuterium dioxide is equally distributed in all liquid body water compartments including saliva”, referring to the IAEA manual. We think this is sufficient to justify the use of salivary samples. If the reviewer would like to suggest a specific reference, we are of course happy to consider that.*

What methods/approach did you use to limit isotopic fragmentation when collecting saliva samples, especially in a likely hot environment?

*RESPONSE: As described on lines 184-189, the samples were stored in a cool box during the study day and transported during the same day to a laboratory freezer.*

Were the analysis of the samples calibrated against standards, e.g. SMOW2 SAP?

*RESPONSE: The Agilent device was calibrated against gravimetrically prepared standards. The precision of the FTIR followed the IAEA recommendation: within and between-day coefficients of variation for the deuterium standard were ≤0.55%. We have added a notion on this on lines 193-195.*

Line 170: A brief description of the method would be welcomed.

*RESPONSE: This is described in the remaining part of the same paragraph.*

Line 174: Please ad that FM was obtained by subtracting FFM to BM.

*RESPONSE: Done.*

Line 177: Please rephrase the first sentence. (“used” instead of “applied”?)

*RESPONSE: Done.*

Please indicate how many of the 240 kids reported to the study visit following your invitation.

*RESPONSE: Added on lines 117-119.*

Provide sample size for each dichotomous variable.

*RESPONSE: There were very few missing values related to the variables in Table1. We have explained these numbers and their handling in the footnote and in the text.*

Line 185: how was multicollinearity tested for?

*RESPONSE: We see no particular need for this in predictive modelling when interpretation of causal relationships is not the main purpose. The variable selection methods we have applied ensure that among a group of mutually correlated variables only one is selected. On the other hand, as the criterion for the best model here is the expected performance in predicting new data (and not making causal inferences), even correlated variables can potentially appear together in the best predictive equation. However, this did not happen in our study.*

Lines 188-189: Please rephrase this sentence.

*RESPONSE: We are not sure how the reviewer would like this sentence to be rephrased. We are happy to reconsider this if the reviewer indicates what is the problem with this sentence.*

Lines 191-192: please rephrase by avoiding future tense.

RESPONSE: Done.

Line 243-244: Why did you decide to only use the RCV predictors? How many equations did you end up getting in total? What was the range of the percentage of the variance that was obtained with those equations?

*RESPONSE: Each of the three model selection approaches (RCV, BIC, AIC) produced one final equation for each outcome (TBW(kg) or TBW(%)) and for the joint model and separate male/female models. RCV was chosen as the estimated predictive performance of the RCV approach was best (measured by lowest average RMSE in repeated cross validation samples).*

*Last column of Table 3 shows the proportion of the variance of the outcome variable accounted by the predicted values.*

Line 217: Please spell out “supplemental”.

*RESPONSE: Done.*

Results

Line 222: Please rephrase the first sentence by modifying “entered”.

*RESPONSE: This has been replaced by “applied in”.*

Table 2: Add data for fat-free mass and body mass.

*RESPONSE: Done.*

In Table 3, it is unclear how TBW in kg was estimated on the first line.

*RESPONSE: We have now added a more descriptive caption for Table 3*.

Table 4: It is unclear what (a), (b) and (c) are referring to, the three lines/models in Table 3?

*

*RESPONSE: We have now added (a),(b),(c) also in Table 3.*

It is unclear why the predictions are worse for TBW in % than in kg when the only difference is the consideration of body mass that was an independent measure.

*RESPONSE: The %-values clearly cannot be very accurately predicted in our data. However, as in our population there is wide variation in body mass, it is inevitable that predicting the TBW(kg)-values at least to some extent is easy. We can always predict that a heavy person has a higher TBW(kg) than a light person even when we cannot predict the actual TBW(%) very accurately.*

*See the answer regarding the %-predictions above.*

Figures 2 and 3 are missing.

*RESPONSE: We apologize that for some reason the LOA plots (Figs 2 and 3) were accidentally not included in the original submission. They have now been included.*

Discussion
**It is difficult to know how generalizable the results of this study are and if this equation can be used for populations other than Malawi kids. Would the authors have possibilities to test the equation of other data sets? We encourage you to further compare your results with those obtained in populations from Senegal, Gambia and Nigeria.**

*RESPONSE: We agree that this would be relevant but would rather see it as a topic of a separate study.*

It would be important to discuss your results on FFM and FM against the clinically significantly difference in children. The bias interval of confidence for TBW being large (+/-4.2% for boys; 3.7% for girls), we can wonder if the BIA method would allow to detect any significant differences across populations or changes.

*We agree that it is important to make clear whether the predictive equations would be useful in a clinical setting, e.g. in an individual level. Indeed the main rationale of our study is to enable a more reliable measurement of body composition in scientific studies on a group level. We have highlighted this by adding a statement regarding the individual-level prediction errors on Lines 376-380.*

The need for electricity on the field in this population maybe a limitation. One can wonder if the skinfold thickness method, albeit less accurate, not be more appropriate for this population.

*RESPONSE: This is correct; our study pertains to settings where electricity can be made available at a research clinic.*
