# Supplementary material for: Body composition among Malawian young adolescents: Cross-validating predictive equations for bioelectric impedance analysis using deuterium dilution method
Source: PLoS One. 2023 Apr 12;18(4):e0284158. doi: 10.1371/journal.pone.0284158 (PMC10096513; doi:10.1371/journal.pone.0284158)
Supplement: Attachment — Submitted filename: renamed_e3298.docx [file pone.0284158.s006.docx]

PONE-D-21-31743R1

Body composition among Malawian young adolescents: Cross-validating predictive equations for bioelectric impedance analysis using deuterium dilution method

PLOS ONE

***Responses to comments by the Academic Editor and Reviewers in and 2***

Comment from Academic Editor: In particular, please take a special attention at the -although minor- important comments of reviewer-2 who pointed again some issues that were raised by both reviewers during the first round of review. This new step of review requires additonal corrections and justifications of some aspects of your study. Please address these thourougly this time and revised your mansucript accordingly.

*RESPONSE: Thank you for your positive response and support. We have taken the remaining requests of reviewer 2 into consideration and modified the manuscript accordingly. A point raised by both reviewers was multicollinearity. We agree that this is important in regression modeling when the role of specific predictors and their coefficients is of the focus of interest. This has typically been the starting point in most bioimpedance studies i.e. variables are selected based on p-values without explicit emphasis on prediction, and the predictive power is then demonstrated using a validation set.*

*However, as we point out to our responses to both reviewers, our approach is to build the model by explicitly optimising expected predictive performance. Shmueli (Statistical Science 2010;25(3): 289-310) writes that multicollinearity in predictive modeling is typically not problematic. Multicollinearity causes additional uncertainty in the individual regression coefficients, but not in predictions produced by the model. Our predictive variable selection approach is parsimonious and will automatically not include variables in the same model if they are severely mutually correlated (then some variables would not possess any additional predictive power in the presence of others). In the selected model, the included predictors may be correlated but nevertheless each is guaranteed to increase the predictive power of the model as variables were selected using the approach optimising predictive performance. We therefore think that the reviewers’ concern has been addressed by the predictive modeling method we use.*

[Note: HTML markup is below. Please do not edit.]

Reviewers' comments:

Reviewer's Responses to Questions

Comments to the Author

1. If the authors have adequately addressed your comments raised in a previous round of review and you feel that this manuscript is now acceptable for publication, you may indicate that here to bypass the “Comments to the Author” section, enter your conflict of interest statement in the “Confidential to Editor” section, and submit your "Accept" recommendation.

Reviewer #1: All comments have been addressed

Reviewer #2: (No Response)

2. Is the manuscript technically sound, and do the data support the conclusions?

The manuscript must describe a technically sound piece of scientific research with data that supports the conclusions. Experiments must have been conducted rigorously, with appropriate controls, replication, and sample sizes. The conclusions must be drawn appropriately based on the data presented.

Reviewer #1: Yes

Reviewer #2: Yes

3. Has the statistical analysis been performed appropriately and rigorously?

Reviewer #1: Yes

Reviewer #2: Yes

4. Have the authors made all data underlying the findings in their manuscript fully available?

The PLOS Data policy requires authors to make all data underlying the findings described in their manuscript fully available without restriction, with rare exception (please refer to the Data Availability Statement in the manuscript PDF file). The data should be provided as part of the manuscript or its supporting information, or deposited to a public repository. For example, in addition to summary statistics, the data points behind means, medians and variance measures should be available. If there are restrictions on publicly sharing data—e.g. participant privacy or use of data from a third party—those must be specified.

Reviewer #1: Yes

Reviewer #2: No

5. Is the manuscript presented in an intelligible fashion and written in standard English?

PLOS ONE does not copyedit accepted manuscripts, so the language in submitted articles must be clear, correct, and unambiguous. Any typographical or grammatical errors should be corrected at revision, so please note any specific errors here.

Reviewer #1: Yes

Reviewer #2: Yes

6. Review Comments to the Author

Please use the space provided to explain your answers to the questions above. You may also include additional comments for the author, including concerns about dual publication, research ethics, or publication ethics. (Please upload your review as an attachment if it exceeds 20,000 characters)

**Reviewer #1**: The authors have largely satisfactorily addressed the issues raised in my original review and have modified the manuscript accordingly.

*RESPONSE: Thank you for your support.*

However, I note that both I and the second reviewer raised the issue of assessing multicollinearity in the regression. The authors have chosen not to pursue this. I am not going to insist upon this point but I am not entirely convinced by their arguments that this should be ignored.

*RESPONSE: As we point out in our response to reviewer 2, we agree that multicollinearity and VIF-values are important in regression modeling when the role of specific predictors and their coefficients is of the focus of interest. This has typically been the starting point in most bioimpedance studies i.e. variables are selected based on p-values without explicit emphasis on prediction, and the predictive power is then demonstrated using a validation set.*

*However, our approach is to build the model by explicitly optimising expected predictive performance. As stated in Shmueli (Statistical Science 25(3): 289-310), multicollinearity in predictive modeling is typically not problematic. Multicollinearity causes additional uncertainty in the individual regression coefficients, but not in predictions produced by the model. Our predictive variable selection approach is parsimonious and will automatically not include variables in the same model if they are severely mutually correlated (then some variables would not possess any additional predictive power in the presence of others). In the selected model, the included predictors are correlated but nevertheless each is guaranteed to increase the predictive power of the model as variables were selected using the approach optimising predictive performance. Reporting VIF-values of predictors would imply that large enough values warrant some caution or action. Using our approach there is no need for this. Therefore we think that reporting VIF-values for the variables would provide no benefit but rather be confusing to the reader.*

Similarly, I am not entirely convinced of the inadequacy of a single split-plot approach to agreement assessment. This approach replicates "real world" measurement where single application of a prediction method in essence reflects a single sample approach and the errors inherent therein. The multi-fold approach as acknowledged by the authors loses knowledge of this variance. But again, opinions vary in statistics as elsewhere!

*RESPONSE: We agree that different resampling methods have been a topic of discussion in literature. We justify our selection of the 10-fold split approach on lines 348-353 of the discussion, with references to some of this discussion.*

**Reviewer #2**: Comments to the authors

The authors have provided with extensive answers to each comment and they made an effort to justify their positions and choices.

However, I still have few minor comments prior to consider this paper ready for publication though. The goal is to improve the quality of the manuscript.

*RESPONSE: We thank the reviewer for support and suggestions for further changes, which we respond to in detail below.*

- Line 241-243: please clarify the methods.

*RESPONSE: We have slightly modified the text trying to make it more clear that our prediction error estimates are based on external data that was not used in model selection. This means that when we select the optimal number of predictor variables through cross validation and find that the minimum average RMSE is achieved for a particular number of predictors, this RMSE value from the modeling stage cannot be used as a reliable error estimate, as it is based on data which is already known to fit best for that particular number of predictor variables. Instead, the RMSE corresponding to the out-sample error should be obtained by predicting new data that was not used in the modeling stage in any manner. The text on lines 235-239 now reads: “Importantly, the RMSE found for the optimal model during the model selection stage was not used in error estimation as this would imply using the same data for modeling and performance evaluation, leading to overly optimistic error estimates [30]. Instead, an out-of-sample prediction error estimate was obtained by predicting data that was not utilised in modeling. For RCV, this is the nested cross validation approach recommended e.g., by Krstajic et al [30].”*

- Line 318-321: Please add p-value and R2 for the associations.

*RESPONSE: The R2-values in Table 3 describe the proportion of variance explained by the out-of-sample predictions and are the squared correlation coefficients in the scatterplots in Fig 2 and 3. We have added these values to the figure legends as suggested by the reviewer. However, these values do not correspond to any testing of a null hypothesis as they are not fitted values from a single regression model (but out-of-sample predictions from 10-fold split of the data). Therefore, we think that reporting p-values would not be appropriate.*

- It is still unclear to me why you could not provide the equations with the 52 subjects who had both empty bladder and who were fasted. This could be shown as supplemental material.

*RESPONSE: As we state in our response to the comments to the first version, results for equation creation and crossvalidation in such a subgroup of 52 of 178 participants (29%) would involve a lot of uncertainty and random error, further aggravated by the fact that self-reported fasting status itself is also uncertain. We believe presenting such equations would be misleading and not appropriate. As we also state in our previous response, we considered separate models for the fasted group (N=90) and for those who did not fast (N=88), but also in these subgroups we noticed that the prediction error is higher and the form of the best performing equation is more uncertain for the non-fasted group.*

*We agree that non-standardised fasting is a limitation. However, this limitation also reflects practical circumstances in conducting studies in low-resource settings. We discuss this in the limitations section, lines 384-386 “As a further limitation, only a half or the participants had fasted overnight, and participants were not systematically asked to empty their bladder after arriving to the clinic, which could increase random error in the measurements.”*

- I am not convinced by the arguments of your choice of height and BMI for the equations. R1 asked to verify collinearity using VIF. You don’t seem to have run this test. I however agree if would be important to do and thus check if your choice of height and BMI is appropriate using a statistical test.

*RESPONSE: We agree that multicollinearity and VIF-values are important in regression modeling when the role of specific predictors and their coefficients is of the focus of interest. This has typically been the starting point in most bioimpedance studies i.e. variables are selected based on p-values without explicit emphasis on prediction, and the predictive power is then demonstrated using a validation set.*

*However, our approach is to build the model by explicitly optimising expected predictive performance. As stated in Shmueli (2010*) multicollinearity in predictive modeling is typically not problematic. Multicollinearity causes additional uncertainty in the individual regression coefficients, but not in predictions produced by the model. Our predictive variable selection approach is parsimonious and will automatically not include variables in the same model if they are severely mutually correlated (then some variables would not possess any additional predictive power in the presence of others). In the selected model, the included predictors are correlated but nevertheless each is guaranteed to increase the predictive power of the model as variables were selected using the approach optimising predictive performance. Reporting VIF-values of predictors would imply that large enough values warrant some caution or action. Using our approach there is no need for this. Therefore we think that reporting VIF-values for the variables would provide no benefit but rather be confusing to the reader.*

*(*) Shmueli: To Explain or to Predict?*

*Statistical Science 25(3): 289-310 (August 2010). DOI: 10.1214/10-STS330*

*As to the specific point of multicollinearity between height and BMI, which the reviewer pointed out in the first version of the manuscript, we refer to our response to the first version: “Among our participants the correlation coefficient between height and weight is 0.87, between weight and BMI 0.84, and between height and BMI 0.47. As expected based on this, our best predictive equation for TBW(%) includes height and BMI. Using just weight instead of BMI would result in inferior predictions. Height being also a component BMI indicates that the dependency on height is not linear and that this nonlinearity is important to be taken into account. In our view, transformations (and interactions) of the original variables can in general be valid potential predictors alongside with the original variables. BMI is a prime example of such a transformed variable.”*

- In the discussion, conclusion and abstract, it needs to be much clearer that this device is appropriate to be used at group/pop level and not at the individual level. Although it has been mentioned, it is still not a clear statement. I would also suggest to run a sample size power analysis using FFM to estimate the sample size needed for such a study in children/adolescents. This will give the reader an idea of how big the study would need to be.

*RESPONSE: We agree and have rephrased sentences in the introduction (lines 71-72): “Balancing accuracy, reproducibility, and ease of measure, BIA is likely the preferred method in field-based* ***population*** *studies [7].” discussion (lines 366-370): “The prediction equations contribute towards meeting the increasing need for affordable but accurate body composition measures for nutrition and health studies in* ***populations of*** *children and adolescents in rural Sub-Saharan Africa, which is essential to understand body composition and its changes in changing nutritional environments” and conclusion (lines 391-394) “These equations enable measurement of body composition by Seca m515 body composition analyser in prepubertal and pubertal adolescents in clinic-based ~~field~~* ***population*** *studies in rural Africa, where electricity is available.” As to running a power analysis at this stage, we respectfully disagree. There is a wide consensus in the medical statistics community that power analyses are a tool of planning studies and the idea of a post hoc power analysis is conceptually flawed and such analyses should not be conducted (see e.g. Jiroutek J Clin Hypertens 2018;20:408-410; Zhang et al. Gen Psychiatry 2019;32:e100069; Lydersen Ann Rheum Dis. 2015;74:323-5.). The power of the study is indicated by confidence intervals.*

- Line 398-402: Please consider adding the fact that the device is expensive and not transportable which will limit studies especially on the field.

*RESPONSE: We have revised the text which now states: “The device* ***is relatively costly*** *and more difficult to transport than a number of portable devices in the market.”*

- In the introduction, the choice of BIA over other available methods is still not clear to me.

*RESPONSE: While thorough comparison of different methods in the introduction would be outside the scope of this manuscript, we have added a sentence “Balancing accuracy, reproducibility, and ease of measure, BIA is likely the preferred method in field-based population studies [7]”*

- I still don’t understand the weight change during the isotopic dilution measurement. What is the reason for this change? In your answer, you stated that weight change was included to estimate any possible isotopic fragmentation. While this is interesting, it seems it would be possible only if you knew the exact value of TBW, which you don’t especially that you assumed (like any other investigator using this method) that you collected urines at the plateau. So I am still having hard time getting the point here.

*RESPONSE: We regret if the response has not made this clear enough, and we have not used exact terminology. Isotopic fractionation is the correct term, not fragmentation. By that we mean that water evaporating in breath or transdermally has a lower deuterium dioxide concentration than body fluids, and evaporation could result in higher deuterium oxide concentrations. (By contrast, after equilibration body fluids such as saliva, urine, faecal water or sweat have similar deuterium oxide concentrations) (Ref 13 IAEA manual). For this reason we have included weight loss during the deuterium dilution test in the modelling procedure as a proxy of water evaporation. Weight loss was, however, not chosen by the modelling procedure.*

*We would also like to emphasise that as per standard procedure, we measured deuterium dioxide concentrations in saliva, not in urine, as the reviewer states.*

7. PLOS authors have the option to publish the peer review history of their article (what does this mean?). If published, this will include your full peer review and any attached files.

If you choose “no”, your identity will remain anonymous but your review may still be made public.

Do you want your identity to be public for this peer review? For information about this choice, including consent withdrawal, please see our Privacy Policy.

Reviewer #1: Yes: Leigh Ward

Reviewer #2: Yes: Audrey Bergouignan

*ADDITIONAL CHANGE*

*To improve the usability of the R code, we have made a minor change to the R code file in supplementary material as follows:*

*File*

*Näsänen-Gilmore-S1-file-R-code for Cross-validating predictive equations (2021_09_17).R*

*Rows 63-68*

*random_split<-function(dd,nf)*

*#random split of data "dd" into "nf" folds of (nearly) equal size*

*{u<-0.5+rank(runif(nrow(dd))); u<-(u-1)/(length(u))*

*limits<-(seq(nf)-1)*(1/nf); folds<-as.list(seq(nf))*

*for (k in 1:nf) {folds[[k]]<-which((u>limits[k])&(u<(1/nf)+limits[k]))}*

*folds}*

*replaced by the following*

*random_split<-function(dd,nf)*

*#random split of data "dd" into "nf" folds of (nearly) equal size*

*{caret::createFolds(seq(nrow(dd)), k = nf)}*
